# Metabolic Response of RAW 264.7 Macrophages to Exposure to Crude Particulate Matter and a Reduced Content of Organic Matter

**DOI:** 10.3390/toxics9090205

**Published:** 2021-08-30

**Authors:** Monika Jankowska-Kieltyka, Adam Roman, Magdalena Mikrut, Marta Kowalska, Rudi van Eldik, Irena Nalepa

**Affiliations:** 1 Department of Brain Biochemistry, Maj Institute of Pharmacology, Polish Academy of Sciences, Smętna Street 12, 31-343 Kraków, Poland; jankow@if-pan.krakow.pl (M.J.-K.); roman@if-pan.krakow.pl (A.R.); marcik48@op.pl (M.K.); 2 Faculty of Chemistry, Jagiellonian University, Gronostajowa Street 2, 30-387 Kraków, Poland; magdalena.mikrut1@gmail.com (M.M.); rudi.vaneldik@fau.de (R.v.E.); 3 Department of Chemistry and Pharmacy, University of Erlangen-Nuremberg, Egerlandstr. 1, 91058 Erlangen, Germany

**Keywords:** particulate matter, cell viability, reactive oxygen species, nitric oxide

## Abstract

Exposure to air pollution from various airborne particulate matter (PM) is regarded as a potential health risk. Airborne PM penetrates the lungs, where it is taken up by macrophages, what results in macrophage activation and can potentially lead to negative consequences for the organism. In the present study, we assessed the effects of direct exposure of RAW 264.7 macrophages to crude PM (NIST1648a) and to a reduced content of organic matter (LAp120) for up to 72 h on selected parameters of metabolic activity. These included cell viability and apoptosis, metabolic activity and cell number, ROS synthesis, nitric oxide (NO) release, and oxidative burst. The results indicated that both NIST1648a and LAp120 negatively influenced the parameters of cell viability and metabolic activity due to increased ROS synthesis. The negative effect of PM was concentration-dependent; i.e., it was the most pronounced for the highest concentration applied. The impact of PM also depended on the time of exposure, so at respective time points, PM induced different effects. There were also differences in the impact of NIST1648a and LAp120 on almost all parameters tested. The negative effect of LAp120 was more pronounced, what appeared to be associated with an increased content of metals.

## 1. Introduction

Exposure to an increased concentration of airborne particulate matter (PM) is considered an important risk factor for human health [1,2]. The problem concerns a large part of the human population, as an increased concentration of PM usually occurs in densely populated areas. Thus, it is not surprising that air pollution with PM is likely to be a public health concern and captures public attention.

PM is a heterogeneous mixture of chemical and biogenic constituents originating from various natural and anthropogenic sources [1,2]. The composition of PM varies substantially across geographical regions, sources of emissions and seasons (e.g., [3,4]). In urban areas, anthropogenic sources prevail because of intense human activities [5]. PM mass is most frequently used as a measure of exposure, but biological effects of PM also depend on the chemical composition and particle size [6]. The roles of specific PM chemical components and mechanisms by which they exert their harmful effects on human health are not completely known yet. The negative effects of PM on human health can be related to specific elements, chemical compounds or their groups, when assessed after both short-term and long-term exposure [7,8]. These data point to the carbonaceous part of PM as being most strongly associated with adverse health effects. Among noncarbonaceous components, several elements were also positively associated with detrimental health effects, namely Ni, V, Fe, Cr, Al, Zn, S, and Pb.

Despite substantial variability in the chemical and physical characteristics of PM, oxidative stress and inflammation are identified as the main factors responsible for cell damage [9,10]. Inflammation is a response of the immune system to a wide range of injurious factors. Airborne PM penetrates into the lungs, where it is taken up by macrophages, resulting in proinflammatory activation of these cells [11]. Because macrophages represent innate immune cells with a potent ability to regulate the immune response [12], their proinflammatory activity may affect the activity of the immune system as a whole. Thus, macrophages represent an important target for negative PM action. Oxidative stress is a metabolic state in which the production of free radicals exceeds the antioxidative capacity of the cell. Both organic and inorganic components of PM can generate ROS directly or via the response of the organism to PM [13]. Most reactive oxygen species (ROS) are generated as a result of impaired respiratory processes in the mitochondria [14]. In phagocytic cells, e.g., macrophages, ROS are also generated by NADPH oxidase, as a mechanism of intracellular killing of ingested microbes [15]. Oxidative stress and inflammation are interconnected because the oxidative status of macrophages is related to their polarization state [16].

In vitro studies are widely used to assess the negative effects of PM on the biological activity of cells due to their simplicity and ease of implementation. The cellular response to PM mainly depends on the PM concentration in the culture medium, chemical composition and physical properties of PM, exposure time, and cell type. Literature reports on the PM effects as a function of exposure time are scarce, and the majority were conducted up to 24 h. However, along with the time of exposure, different cell responses to PM can be observed [17]. It is also known that organic and inorganic components activate different metabolic processes leading to oxidative stress and inflammation [18]. Thus, the toxicity of PM, which has a mixed composition, may differ substantially from the effect of a single ingredient, and an interaction between the components does exist. Moreover, the cytotoxicity of PM components is not always associated with specific health effects [19]. Therefore, in the present study, we assessed the effects of direct exposure of macrophage cells to crude PM and to a reduced content of organic matter on selected parameters of metabolic activity at various exposure times (up to 72 h).

## 2. Materials and Methods

### 2.1. Reagents

Dulbecco’s modified Eagle’s medium (DMEM), fetal bovine serum (FBS), and Dulbecco’s phosphate buffered saline with calcium and magnesium (PBS) were obtained from Gibco (Invitrogen, Poisley, UK). The LDH Cytotoxicity Detection Kit was purchased from Roche (Roche Diagnostics, Indianapolis, IN, USA), while crystal violet from Fluka (Basel, Switzerland). Methanol, potassium hydroxide, dimethyl sulfoxide (DMSO), formaldehyde, and phosphoric acid were obtained from POCh (Gliwice, Poland). Resazurin, propidium iodide (PI), sulfanilamide, N-(1-naphthyl) ethylenediamine dihydrochloride, 2′,7′-dichlorodihydrofluorescein diacetate (DCFH-DA), nitrotetrazolium blue chloride (NBT), and RNAse were purchased from Sigma-Aldrich (St. Louis, MO, USA). NIST1648a was obtained from the National Institute of Standards and Technology (Gaithersburg, MD, USA). Culture dishes were purchased from Corning (New York, NY, USA) and Nunc (Roskilde, Denmark).

### 2.2. PM Treatment and Characterization

NIST1648a is a high-quality reference material of urban air pollution collected in St. Louis. The elemental composition of this dust was fully analyzed and provided on the Certificate of Analysis supplied with the dust [20]. The Plasma Zepto system (Diener Electronic GmbH, Ebhausen, Germany) was used for the removal of organics. Samples of dust were treated with a low-temperature plasma for up to 120 min (referred to as LAp120), and the decrease in the content of organic carbon was monitored using an elemental analyzer (Elementar, Vario Micro Cube) and a total organic carbon analyzer (Shimadzu, TOC-V series equipped with the Total Nitrogen accessory) [21]. Morphological characterization of NIST1648a- and plasma-treated samples was performed by applying scanning electron microscopy (SEM), with a Tescan Vega3 LMU microscope equipped with an LaB_6_ cathode and EDS detector (Oxford Instruments, X-act, Silicon Drift Detector, SDD 10 mm^2^).

### 2.3. Cell Culture and Treatment

The RAW 264.7 mouse macrophage cell line (obtained from the Department of Immunology, Jagiellonian University Medical College, Kraków, Poland) was cultured in DMEM supplemented with 10% heat-inactivated FBS, 50 μg/mL penicillin, and 50 μg/mL streptomycin under standard conditions (37 °C, 90% humidity, 5% CO_2_). The cells were suspended in the culture medium at a concentration of 2 × 10^5^ cells per ml. The cell suspension was dispensed into 96-well flat-bottomed culture plates in volumes of 0.1 mL, and the cells were let to stabilize for 4–6 h.

NIST1648a and LAp120 were suspended in PBS, and the suspensions were sonicated for 3 min in an ultrasonic water bath before being added to cell cultures at a volume of 10 μL per well. The suspensions of NIST1648a or LAp120 were added to obtain final concentrations of 1, 10, and 100 µg per ml of culture medium (which correspond to 0.33, 3.33, and 33.33 μg/cm^2^ of the bottom surface of the well, respectively) at specific points of time before termination of the culture. Control cultures were supplemented with PBS.

### 2.4. Viability Assays

PI uptake by the cells was used as a measure of cell viability, as described previously [22]. Such supravital staining allows us to distinguish between viable, apoptotic, and dead cells [23]. Briefly, at the end of the culture period, cells were washed with PBS and transferred into cytometric tubes, and a solution of PI was added to reach a final concentration of 50 µg/mL. After 10 min of incubation in the dark, the cells were measured in a FACSCanto II flow cytometer using the FACSDiva software (both Becton Dickinson, San Jose, CA, USA). PI fluorescence intensity was measured in the FL-3 channel with an excitation wavelength of 488 nm and emission wavelengths of 670–735 nm. Nonfluorescent cells were gated as viable, and the results are presented as a percentage of all events. Dimly fluorescent cells were gated as late apoptotic cells, and bright cells were considered dead [23]. Viability assessments were carried out after 6, 12, 24, 48, and 72 h of incubation with previously specified concentrations of NIST1648a or LAp120.

Apoptosis was assessed in fixed cells treated in the same way, according to the method described by Nicoletti et al. [24]. In this method, nuclei of fixed cells are stained with PI and the DNA content is measured using flow cytometry. Apoptotic events with reduced DNA content are detected on DNA histograms as so-called sub-G1 peaks. Briefly, at the end of the culture period, the cells were washed in warm PBS and were fixed in 70% cold ethanol for at least 24 h at 4 °C. Afterward, the cells were washed twice in PBS and stained with 150 μg/mL of PI (final concentration) in the presence of RNAse (250 μg/mL) for 30 min. The cells were then measured in a FACSCanto II flow cytometer using FACSDiva software (both Becton Dickinson, San Jose, CA, USA). The threshold was set on PI fluorescence and doublets were excluded on the basis of a pulse area to pulse width ratio [25]. The assessment was conducted after 6, 12, 24, 48, and 72 h of incubation with previously specified concentrations of NIST1648a or LAp120.

The lethality of cells in culture was estimated on the basis of lactate dehydrogenase (LDH) activity in the culture medium using the LDH detection kit (SigmaAldrich St. Louis, MO, USA) [22] according to the manufacturer’s instructions. Briefly, 50 μL of supernatant was mixed with 50 μL of PBS in 96-well flat-bottomed microplates. The LDH detection kit solutions were mixed in an appropriate ratio and promptly added to 100 μL of the diluted supernatants at a volume of 100 μL. The plates were incubated in the darkness for 30 min. Afterward, the absorbance was measured at 492 nm, with a reference wavelength of 620 nm, using a Synergy MX microplate reader working under the control of the Gen5 software (both BioTek Instruments, Winooski, VT, USA). The LDH test was conducted immediately after treatment with PM (time point 0 h) and after 4, 24, 48, and 72 h of incubation with specified concentrations of NIST1648a or LAp120. The data were expressed as the percentage of the values observed in control cultures neither exposed to NIST1648a nor LAp120.

### 2.5. Assessment of Metabolic Activity

The overall metabolic activity of the cells was assessed using the resazurin reduction assay, as described previously [26]. Resazurin is a redox-sensitive compound that is reduced mainly by the mitochondrial respiratory chain, and its reduced form is released into the culture medium [27]. In brief, after specified incubation periods with PM, 10 μL of resazurin solution (0.44 mM in PBS) was added to each well of the culture plates. After 1.5 h of incubation under standard conditions, fluorescence (excitation and emission wavelengths were 560 and 590 nm, respectively) was measured using a Synergy MX microplate reader working under the control of the Gen5 software (both BioTek Instruments, Winooski, VT, USA). The data were presented as the percentage of the values observed in control cultures.

### 2.6. Detection of Reactive Oxygen Species

Reactive oxygen species (ROS) were detected using a cell-permeable fluorogenic probe DCFH-DA and a microplate reader [28]. DCFH-DA is responsive to several reactive oxygen species, and such a nonspecificity of the probe allows us to assess the general oxidative capacity of PM rather than selected ROS production [29]. Briefly, after specified incubation periods with PM, the cell cultures were washed with PBS, and 10 μL of 50 µM DCFH-DA was added to each well. Then, the plates were incubated under standard conditions for 90 min, and fluorescence (excitation and emission wavelengths were 492 and 520 nm, respectively) was measured using a microplate reader. The data were expressed as the percentage of the values observed in control cultures.

ROS synthesis was also assessed at the single-cell level in viable cells using flow cytometry of double-stained cells with DCFH-DA and PI, according to the method described by Sureda et al. [30]. Briefly, RAW 264.7 cells suspended in PBS were placed in cytometric tubes in a volume of 0.1 mL and NIST1648a or LAp120 were added at a previously specified concentration. Immediately after PM treatment (time point 0 h) and after 1 or 4 h of incubation under standard culture conditions, 10 μL of 50 µM DCFH-DA was added to each tube and incubation continued for the subsequent 30 min. Then, the cells were supravitally stained with PI, as described previously, and assessed using a cytometer. DCFH-DA and PI fluorescence intensity were measured in the FL-1 and FL-3 channels, respectively. Viable cells were gated within single cells (discriminated on the basis of forward scatter [25]) as PI negative, and in this cell population, DCFH-DA fluorescence was assessed and expressed in relative fluorescence units (RFU).

### 2.7. Assessment of Cell Number

Cells were stained with crystal violet (CV), as described elsewhere [31]. Under these conditions, absorbance is proportional to cell number [32]. Briefly, immediately after treatment with NIST1648a or LAp120 and following a 4, 24, 48, and 72 h incubation with specified concentrations of NIST1648a or LAp120, 100 μL of 2% formaldehyde was added to each well (yielding a 1% final concentration) and the cells were fixed for 1 h at room temperature. Afterward, supernatants were removed, and the cells were stained with 50 μL 0,5% CV dissolved in 20% methanol for 5 min. The plates were then washed three times with deionized water and the absorbed dye was extracted with 100 μL of 100% methanol per well. The absorbance was immediately measured at 570 nm using a microplate reader. The data were expressed as percentages of the values observed in control cultures.

### 2.8. Assessment of Nitric Oxide Release

Nitric oxide (NO) synthesis was evaluated as the accumulation of nitrites in the culture medium during a specified incubation period using the Griess’s reaction, as described previously [31]. PM-induced NO release was assessed only at three time points, 24, 48, and 72 h after the addition of NIST1648a or LAp120 to the cell cultures, because it requires gene expression and protein synthesis of inducible nitric oxide synthase (iNOS). An increase in nitrites in the culture medium is clearly observed after 24 h of culture [33]. Briefly, 50 μL of supernatant was mixed with 30 μL of 1% sulfanilamide dissolved in 5% phosphoric acid and 30 μL of 0.1% *N*-(1-naphthyl)ethylenediamine dihydrochloride dissolved in water. The absorbance was measured at 540 nm using a microplate reader and was expressed as percentages of the control values.

### 2.9. Assessment of Respiratory Burst

The ability to synthesize the superoxide anion (O_2_^-^) in a process called respiratory burst was assessed using the NBT reduction test, as described previously [31]. This method allows us to assess the cytotoxic activity of macrophages, the cells of innate immunity [34]. In brief, immediately after treatment with NIST1648a or LAp120 and following a 4, 24, 48, and 72 h incubation with specified concentrations of NIST1648a or LAp120, 10 μL of 1% aqueous NBT solution was added per well, and the incubation continued for 2 h. Next, the supernatants were gently removed, and the wells were washed three times with 100% methanol. Accumulated formazan was dissolved by adding 120 μL per well of 2 M potassium hydroxide and 140 μL per well of DMSO, and the mixtures were vigorously mixed with a multi-pipette. Then, the absorbance was measured at 630 nm using a microplate reader. The data were expressed as percentages of the values observed in control cultures.

### 2.10. Statistical Analysis

All assays were carried out in at least two replicates and repeated at least two times. The data presented in the figures represent the results obtained in one of the experiments. Statistica 10.0 for Windows (Statsoft, Tulsa, OK, USA) was used for the analysis of data, which are reported as the means ± standard error of means (SEM). The normality of variable distributions and homogeneity of variances were verified by the Shapiro–Wilk and Levene’s tests, respectively. Data were evaluated by a two-way analysis of variance (ANOVA) with PM (NIST1648a or LAp120) × concentration as the factors, separately for each time point, and by planned comparisons (contrast estimation). When ANOVA assumptions were not fulfilled, the Kruskal–Wallis ANOVA (by ranks) for multiple comparisons was used. *p*-values lower than 0.05 were regarded as significant.

## 3. Results

### 3.1. NIST1648A and LAp120 Composition

Organic carbon compounds were removed from NIST1648a by oxidation, applying oxygen plasma treatment. Elemental analyses indicated a decrease in the amount of carbon from 14.10% to 1.8% after 2 h of plasma treatment. This result was confirmed by a total organic carbon analysis, which showed that both the original NIST1648a and that subjected to 2 h of plasma treatment (LAp120) included almost exclusively organic carbon (Table 1). Additionally, the hydrogen and nitrogen contents decreased, which can be related to the oxidation of polycyclic hydrocarbons and nitro-substituted polycyclic hydrocarbons. Only the sulfur content increased, what may result from decreasing the overall mass of the sample upon plasma treatment, and not removing sulfur-containing compounds.

**Figure 1:**
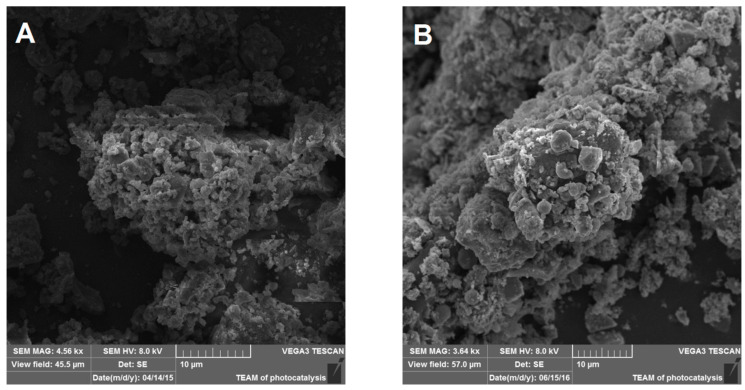
NIST1648a sample before (**A**) and after 2 h of plasma treatment (**B**).

The morphology of the treated dust was analyzed using scanning electron microscopy (Figure 1). Aggregates of particles were present in NIST1648a and LAp120. It seems that the plasma treatment did not affect the morphology of the samples.

### 3.2. Effect of PM on Cell Viability

**Figure 2:**
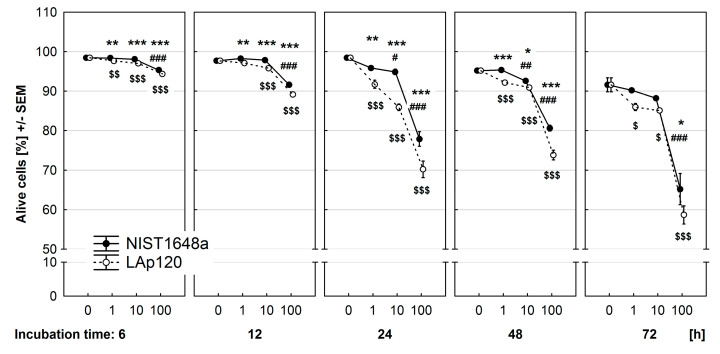
Effect of PM exposure on cell viability assessed using supravital propidium iodide (PI) staining and flow cytometry and expressed as % of live cells (not stained with PI) among all events. RAW 264.7 cells were exposed to NIST1648a or LAp120 at concentrations ranging from 0 to 100 μg/mL of culture medium (abscissa) for 6–72 h. #, ##, ###—*p* < 0.05, 0.01, 0.001, respectively, relative to cultures not exposed to NIST1648a (PM 0 μg/mL) at the same time point; $, $$, $$$—*p* < 0.05, *p* < 0.01, 0.001, respectively, relative to cultures not exposed to LAp120 (PM 0 μg/mL) at the same time point; and *, **, ***—*p* < 0.05, 0.01, 0.001, respectively, for cultures exposed to NIST1648a vs. LAp120 at the same concentration and time point.

The viability of RAW 264.7 cells exposed to selected concentrations of NIST1648a or LAp120 for 6–72 h was measured using supravital staining with PI and flow cytometry, and the results are presented in Figure 2. In control cultures, the viability of the cells was very high (over 95%) and decreased after 48 and 72 h. Both NIST1648a and LAp120 significantly (*p* < 0.001 at each time point) decreased the viability of the cells in a concentration-dependent manner, and this effect was substantially intensified with extending exposure time. Interestingly, the effect of LAp120 was more pronounced than that induced by NIST1648a at each time point (*p* < 0.05). Negative effects of the highest concentration of both PM forms were visible at all time points. NIST1648a at a concentration of 10 μg/mL decreased cell viability after 24 and 48 h of exposure, whereas LAp120 at concentrations of 1 and 10 μg/mL decreased cell viability at almost all time points.

As a result of exposure to PM, an increase in the percentage of apoptotic and dead cells in cultures was observed. In control, unexposed cultures, spontaneous cell mortality was very low (below 2%) and increased from 48 h on (Figure S1 in the Supplementary Material). The increase in the percentage of dead cells due to PM exposure was concentration-dependent (*p* < 0.001 at each time point) and intensified with the extension of exposure time. The effect of LAp120 was more pronounced than that induced by NIST1648a at each concentration and time point (*p* < 0.05).

The application of supravital staining of the cells with PI and flow cytometry enabled detection and assessment of late apoptosis. Similarly to previously described results, in control cultures, spontaneous cell apoptosis was very low (below 2%) and increased starting from 48 h (Figure S2 in the Supplementary Material). The increase in the percentage of apoptotic cells due to PM exposure was significant at all time points (*p* < 0.001 as assessed with the Kruskal–Wallis test) and intensified with the extension of exposure time only at the highest concentration used. The effect of LAp120 was more pronounced than that of NIST1648a; however, the differences were relatively small and statistically significant after 12 and 24 h of exposure.

Apoptotic cell death was also assessed in fixed cells stained with PI using flow cytometry (Figure S3 in the Supplementary Material). This method detects the previous stage of apoptosis, when DNA fragmentation occurs. Spontaneous cell apoptosis detected with this method was somewhat different (4–7%) from that assessed using supravital staining with PI. NIST1648a at the highest concentration significantly (at *p* < 0.001) increased the percentage of sub-G1 events, starting from 12 h of exposure. Lower concentrations of NIST1648a exerted no effect or even decreased apoptosis after 6 and 72 h of exposure (*p* < 0.01). Exposure of the cells to LAp120 generally decreased apoptosis detected with this method, except for cultures exposed to the highest concentration for 24 and 48 h (*p* < 0.01 and 0.001, respectively).

Additionally, the lethality of the cells was also measured using the LDH test, and the results are presented in Figure S4 in the Supplementary Material. The assessments were carried out at slightly different time points, namely, immediately after PM treatment and following 4, 24, 48, and 72 h of exposure. At the first two time points, no significant changes in LDH activity were found. A clear negative effect of the highest concentration of NIST1648a and LAp120 appeared starting from 24 h of exposure (*p* < 0.01). The negative effect of LAp120 was more pronounced but reached statistical significance after 72 h of exposure.

### 3.3. Effect of PM on Metabolic Activity

**Figure 3:**
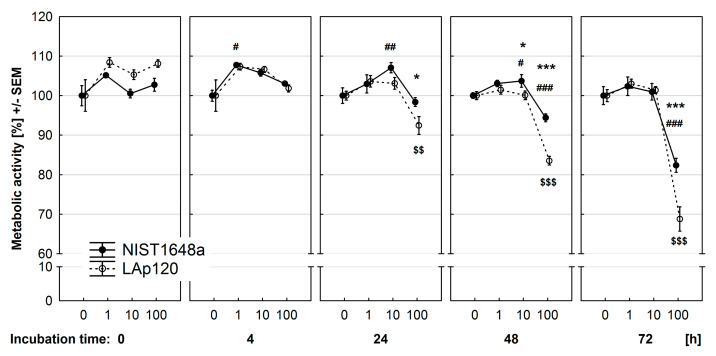
Effect of PM exposure on metabolic activity of the cells assessed using the resazurin reduction test and expressed as % of the values observed in control cultures not exposed to PM. RAW 264.7 cells were exposed to NIST1648a or LAp120 at concentrations ranging from 0 to 100 μg/mL of culture medium (abscissa) for 0–72 h. Significances as in Figure 2.

The overall metabolic activity of the RAW 264.7 cells was assessed using the resazurin reduction test, and the results are presented in Figure 3. Both forms of PM, especially LAp120, at all concentrations used, statistically enhanced the metabolic activity of the cells when assessed immediately after addition to the culture. NIST1648a at the highest concentration significantly (*p* < 0.001) decreased this parameter after 48 and 72 h of exposure. A similar effect was caused by LAp120 at the same concentration, but this effect occurred earlier, also after 24 h of exposure (*p* < 0.01), and was significantly more severe (*p* < 0.001 and *p* < 0.01). Lower concentrations of both PM forms, in contrast to the highest one, increased metabolic activity after 4, 24, and 48 h of exposure.

### 3.4. Effect of PM on ROS Synthesis

**Figure 4:**
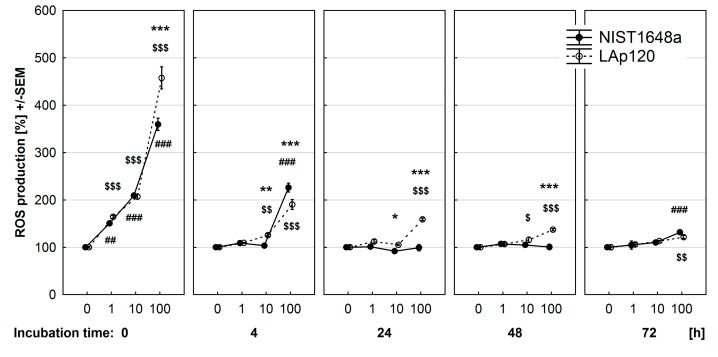
Effect of PM exposure on ROS generation assessed using a fluorescent probe DCFH-DA and expressed as a percentage of the values observed in control cultures not exposed to PM. RAW 264.7 cells exposure conditions and significances as in Figure 3.

As shown in Figure 4, both NIST1648a and LAp120 resulted in a substantial and concentration-dependent increase in ROS formation directly after addition to the cultures. The stimulatory effect of LAp120 was higher than that of NIST1648a only at the highest concentration used (*p* < 0.001). At subsequent time points, the stimulatory effect of LAp120 gradually diminished, and after 72 h of exposure, it was still significant at the highest concentration (*p* < 0.01). The stimulatory effect of NIST1648a also decreased at the 4 h time point (*p* < 0.001 for the highest concentration used) and declined after 24 and 48 h of exposure but appeared again (*p* < 0.001) at the last time point.

The above-mentioned results were obtained with a microplate reader, used to assess ROS synthesis in cultures at the whole well level. It can be assumed that the reduction in ROS synthesis produced by NIST1648a or LAp120 exposure may be due to increasing cell mortality, as evidenced by previously presented results. Thus, we conducted an additional assessment of ROS synthesis at the single-cell level using flow cytometry after short-term exposure (up to 4 h of incubation) to PM (Figure S5 in the Supplementary Material). Viable cells were distinguished on the basis of PI uptake. It was shown that the synthesis of ROS by single viable cells was the highest immediately after the addition of PM to the culture medium and decreased with incubation time. Both NIST1648a and LAp120 increased ROS production directly after addition to the cell cultures in a concentration-dependent manner, and this effect decreased with exposure time (*p* < 0.001 at each time point). The effect of LAp120 was significantly stronger than that of NIST1648a (*p* < 0.001 at each time point). NIST1648a-induced ROS synthesis decreased faster than the one induced by LAp120, and after 4 h of exposure, ROS synthesis was even lower than in control cells.

### 3.5. Effect of PM on Cell Number

The effect of NIST1648a and LAp120 on the number of cells in the cultures evaluated by CV staining is shown in Figure S6 in the Supplementary Material. NIST1648a did not change the number of cells in the cultures assessed immediately after the addition to culture medium and after 4 h of exposure. At the subsequent time points, only the highest concentration resulted in a significant reduction in cell number (*p* < 0.01, *p* < 0.001, and *p* < 0.001, respectively). The negative effect of LAp120 at the highest concentration appeared earlier, after 4 h of exposure, and was significantly more pronounced than that of NIST 1648.

### 3.6. Effect of PM on NO Release

**Figure 5:**
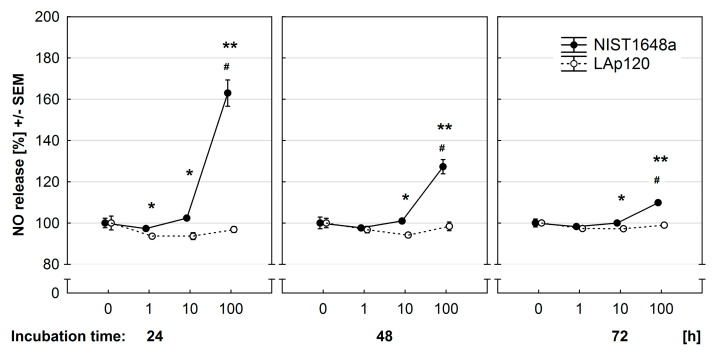
Effect of PM exposure on nitric oxide (NO) synthesis assessed using the Griess’ reaction and expressed as a percentage of the values observed in control cultures not exposed to PM. RAW 264.7 cells were exposed to NIST1648a or LAp120 at concentrations ranging from 0 to 100 μg/mL of culture medium (abscissa) for 24–72 h. Significances as in Figure 2.

In the present study, the nitrite level in the culture medium was relatively low, but changes induced by NIST1648a were clearly visible. The obtained results are presented in Figure 5. At all three time points studied, NIST1648a at the highest concentration used increased NO release by RAW 264.7 cells (*p* < 0.01), whereas LAp120 did not. NO release diminished with exposure time. Differences between the effects of both PM forms were statistically significant at all time points not only for the highest concentration (*p* < 0.01) but also for a concentration of 10 μg/mL (*p* < 0.05).

### 3.7. Effect of PM on Respiratory Burst

The PM-induced respiratory burst was the highest immediately after addition to the culture medium and decreased with exposure time (Figure S7 in the Supplementary Material). Both NIST1648a and LAp120 increased the respiratory burst up to 48 h of exposure and decreased it at the last time point studied (*p* < 0.01 at each time point). The stimulatory effect was evoked only by the highest concentration of both PM forms during the first three time points (at *p* < 0.5). After 48 h of exposure, only the effect of NIST1648a was statistically significant (*p* < 0.05). After 72 h of exposure, both PM forms reduced the respiratory burst.

## 4. Discussion

In the present study, we assessed the effects of crude PM material, NIST1648a, and that with a reduced organic component content, LAp120, on common parameters of cell viability, metabolic activity, ROS synthesis, and inflammatory activity of RAW 264.7 macrophages. The obtained results showed that both NIST1648a and LAp120 negatively influenced the parameters of cell viability and metabolic activity under study due to increased ROS generation. The negative effect of PM was concentration-dependent and most pronounced for the highest applied concentration of 100 μg/mL. The impact of PM also depended on the time of exposure, and at the respective time points, PM evoked different effects. There were also differences in the impact of NIST1648a and LAp120 on almost all parameters tested.

A decrease in cell viability is frequently reported as the outcome of exposure of various cell types to PM (e.g., [17,35]). In the present study, both NIST1648a and LAp120 reduced cell viability in culture in a concentration- and time-dependent manner similar to that reported by Michael and coworkers [36]. However, the toxicity of NIST1648a and LAp120 was considerably lower than the one of PM samples from urban and rural areas tested by these authors. Cell viability during culture significantly influences other parameters of metabolic activity under study, so two methods were used for the estimation of cell viability. During supravital staining with PI, we observed increased uptake of PI by cells, which is indicative of decreased cell viability, depending on the concentration of PM and exposure time. Concurrently, an increase in the percentage of dead cells, brightly stained with PI, was observed. This process started after 6 h of exposure to the highest concentration of both PM forms. However, LDH activity in the culture medium, which was another measure of cell viability, increased after 24 h of exposure. Similar results were reported by Deng et al. [37]. In their study, an increase in LDH activity started after 24 h of exposure to the same concentration of PM in the culture medium. This difference may result from the different mechanisms of both detection methods used and various time points at which the study was performed. Supravital PI staining with cytometric evaluation is a very sensitive method that allows for the detection of the late stages of cell death in individual cells [23]. An increase in LDH activity in the culture medium is associated with necrotic death of the cells, and this method enables the evaluation of cell death at the level of the whole culture well [38]. Similar differences between the results of viability assessments using different methods were reported by Kim and coworkers in the H_2_O_2_-induced cytotoxicity of RAW 264.7 cells, where cell death was assessed applying the trypan blue exclusion method and the LDH test [39]. In addition, viability results obtained using the LDH test may be obscured by the interaction of PM with LDH. It has been shown that titanium dioxide (TiO_2_) nanoparticles reduce the activity of this enzyme in the supernatant [40], and a similar phenomenon was also observed for copper and silver nanoparticles [41].

A decrease in the viability of cells exposed to PM is also associated with the induction of apoptosis as a result of increased ROS synthesis. Such effects are frequently reported in the literature (e.g., [42,43]). In the present study, apoptosis was determined by two methods. Supravital staining of the cells with PI enables the detection of late apoptosis on the basis of increased permeability of the cell membrane [23]. With this method, it was shown that PM at the highest concentration induced apoptosis just after 6 h of exposure, and LAp120 exerted a stronger effect than NIST1648a. The study of DNA content in fixed cells [24] allows for the assessment of apoptosis in the earlier phase, when DNA fragmentation occurs. With this method, slightly different results were obtained. Paradoxically, exposure to LAp120 even decreased apoptosis assessed as the percentage of events with sub-G1 DNA content. This effect may be related to rapid necrotic cell death induced by LAp120 in some cells after short-term exposure because the DNA profiles of necrotic and live cells assessed with this method look rather similar [44]. Similar acute effects for short-term exposure of the cells to PM were observed by others (e.g., [45,46]).

The results of cell viability are in line with decreased metabolic activity and decreased cell number in the cultures. Metabolic activity was measured as the ability of the cells to reduce resazurin. This compound is reduced mainly by the mitochondrial respiratory chain and may reflect the overall metabolic activity of the cells in relation to respiratory processes [27]. In our study, an increase in resazurin reduction was observed shortly after treatment of the cells with both PM forms, especially at lower concentrations. This effect may be related to increased oxygen consumption due to elevated ROS synthesis in mitochondria and NADPH oxidase [47]. With the extension of exposure time, metabolic activity decreased, especially in cultures treated with the highest concentration of both PM forms, mainly due to increased cell mortality, and thus decreased cell number in the cultures, as detected using CV staining. These changes may be related to PM-induced disruption of mitochondrial structure and function [48] and increased ROS synthesis and oxidative stress [35].

PM-induced oxidative stress has been regarded as a crucial mediator of PM toxicity [13,49]. Literature reports consistently describe an increase in PM-induced ROS synthesis, although the kinetics of the changes were variable. For example, Deng et al. [37] reported the highest ROS synthesis in the 4th hour of exposure, a decrease in the 12th hour and normalization at subsequent time points. In turn, Piao and coworkers [43] observed the highest ROS synthesis in the 1st hour of exposure with a gradual decrease up to 24 h, when it was still significantly increased. Others noted a gradual increase from the 1st up to the 12th hour with a decrease after 24 h of exposure [50] or a gradual increase in ROS synthesis from the 2nd up to the 48th hour [51] and even up to the 96th hour of exposure [46]. The differences in PM-induced ROS synthesis may be related to various cell lines used, different concentrations of PM as well as physical properties and chemical composition of PM. In the present study, we showed that PM exposure resulted in concentration-dependent immediate ROS synthesis, which rapidly decreased over several hours but remained visible after 72 h of exposure. As was the case with cell viability, metabolic activity and cell number assessment, the effect of LAp120 was more pronounced than that of NIST1648a.

The above-mentioned results were obtained at the whole culture level, and the decrease in ROS synthesis along the exposure time, observed in our study, was caused in part by an increase in cell mortality. By cytometric evaluation of ROS synthesis in single live cells, we confirmed the highest ROS synthesis immediately after the addition of PM to the cultures and its rapid decrease within 4 h. Such an immediate rise in ROS synthesis seems to be responsible for subsequent cell death and a decrease in metabolic activity and other parameters under study. Again, the effect of LAp120 was more pronounced than NIST1648a. Interestingly, in cultures exposed to NIST1648a, slightly, but significantly lower ROS synthesis was observed in live cells after 4 h of exposure. This may be related to the interaction among PM components in the potency to generate ROS, namely, some organic components and metals. This phenomenon was observed when ROS-generating capacity was assessed using the dithiothreitol (DTT) assay in an abiotic system [52]. Similarly, an interaction was reported between some metals and components of bioaerosol included in PM [53]. Because the results obtained with the DTT assay positively correlate with the ability to induce cellular oxidative stress responses [54,55], such interaction may be involved in the lower ROS synthesis after exposure to NIST1648a observed in our study. This presumption seems to be corroborated by the fact that the decrease was observed only in cultures exposed to NIST1648a, which contains the whole spectrum of organic components, but was absent in cultures exposed to LAp120 in which the organic matter content was reduced by cold plasma treatment.

In the present study, we assessed two parameters of macrophage activity related to their function as cells of innate immunity, namely, respiratory burst and NO release. These two manifestations of cell activity occur as a consequence of pathogenic agent action or tissue damage. As a result, macrophages undergo proinflammatory activation, which aims to eliminate pathogens and damaged tissues and, at a later stage, restore the homeostasis of the body.

The respiratory burst is a very fast cell response to stimulation with extrinsic agents, e.g., phagocytosis [56]. During this process, constitutively expressed NADPH oxidase is activated, resulting in a sharp increase in O_2_^−^ synthesis, which is used by phagocytes for intracellular killing of engulfed microorganisms [16]. Exposure of macrophages to PM results in phagocytosis of the particles [57] and subsequent respiratory burst [58]. Both organic [59] and inorganic components, including some metals [58,60], can induce this process.

In the present study, both forms of PM at the highest concentration increased respiratory burst immediately after addition to the culture medium, and the stimulatory effect decreased with time of exposure, probably due to increased cell death. Similar rapid (within 2 h) induction of respiratory burst by PM exposure was observed by others [58,59]. However, PM pre-exposure substantially impaired the respiratory burst induced by typical stimulants: zymosan, phorbol-12-myristate-13-acetate (PMA), and lipopolysaccharide plus interferon gamma [58].

NO released by macrophages is used for intracellular killing of engulfed microorganisms, similar to ROS generated as a result of NADPH oxidase activity [61]. NO is also a modulator of the immune response, and intense NO synthesis is considered a marker for the activity of proinflammatory M1-type macrophages [12]. The results obtained in the present study indicate that NIST1648a induces NO synthesis in macrophages, whereas LAp120 does not. Similarly to the results obtained by Imrich et al. [62], in our study, a NIST1648a-induced rise in the concentration of nitrites in the culture medium was relatively low but statistically significant. This indicates that NIST1648a, in contrast to LAp120, directly stimulates the proinflammatory activity of macrophages. Similar results were obtained by others [63], who showed that NIST1648a, but not LAp120, induced synthesis of proinflammatory cytokines: TNF-α, IL-6, and IL-12p40. The induction of NO synthesis by NIST1648a may be due to the presence of an organic component, namely, bacterial endotoxins that stimulate proinflammatory activity of the cells and the release of proinflammatory cytokines [64]. Similar proinflammatory effects are exerted by some other organic PM components, such as polycyclic aromatic hydrocarbons (PAHs) [65]. The reduction in NO synthesis observed in this work along with the exposure time, may result from an increase in cell death and from decreased expression of iNOS [66].

Although both forms of PM negatively influenced all parameters of cellular activity under study, the effects of LAp120 were consistently more pronounced than those evoked by NIST1648a at the same concentrations. Compared with NIST1648a, LAp120 contains far less organic matter as a result of cold plasma treatment [21] and thus a higher percentage of various biologically active inorganic components, including metals. The content of metals in PM often correlates positively with cell death and the ability to cause oxidative stress in cells [67,68]. NIST1648a used in the present study contains significant amounts of metals, mainly iron (Fe) 3.92%, lead (Pb) 0.66%, zinc (Zn) 4800 mg/kg, titanium (Ti) 4021 mg/kg, manganese (Mn) 790 mg/kg, copper (Cu) 610 mg/kg, chromium (Cr) 402 mg/kg, vanadium (V) 127 mg/kg, arsenic (As) 115.5 mg/kg, and nickel (Ni) 81.1 mg/kg [20]. Apart from organic components, these metals were shown to strongly correlate with oxidative stress and/or cytotoxicity [69,70].

In addition to the chemical composition of PM, the physical characteristics of the particles, mainly size and shape, also determine their bioreactivity. It has been shown that PM fractions of various sizes have different chemical compositions and are characterized by differentiated bioreactivity [70]. As demonstrated in this work, cold plasma treatment did not affect the morphology of PM. It can therefore be concluded that larger bioreactivity of LAp120 is due to changes in the chemical composition. As a result of treatment with cold plasma, the content of these metals in LAp120 increased and was even higher during this process than in NIST1648a (approximately 35% more than in NIST1648a), while the sample mass decreased to approximately 66%. Thus, higher metal contents may be related to a stronger biological effect exerted by LAp120 in comparison with NIST1648a. Differences in the intensity of the biological effects of NIST1648a and LAp120 observed in the present study may also result from the above-mentioned interactions between some organic NIST1648a components and metals [52].

## 5. Conclusions

The results presented in this study indicate that both forms of PM negatively affect viability and metabolic processes of cells. This negative effect is related to elevated ROS synthesis and the induction of oxidative stress. Higher activity of LAp120 compared with NIST1648a appears to be associated with an increased content of metals, which relatively elevates by organic components removal. Our results also showed that 2 h of cold plasma treatment is a good method for the removal of organic matter, including biologically active compounds, such as lipopolysaccharides, which may obscure the effects of inorganic air pollution components containing transition metals and is significant for the APARIC project (“**A**ir **P**ollution versus **A**utoimmunity: **R**ole of multiphase aqueous **I**norganic **C**hemistry”—APARIC) [71].

## Figures and Tables

**Table 1:** Concentration of four elements in NIST1648a and LAp120 samples. Concentration in wt%.

Material	Elemental Analysis				Total Organic Component Analysis			
	C	H	N	S	TC ^1^	IC ^1^	TOC ^1^	TN ^1^
NIST1648a	14.10	2.41	3.12	4.96	9.21	0.09	9.12	2.75
LAp120	1.87	1.20	0.83	6.18	1.75	0.07	1.68	0.22

^1^ TC—total carbon, IC—inorganic carbon, TOC—total organic carbon, TN—total nitrogen.

## Data Availability

The data presented in this study are available in the article or in the supplementary material.

## Supplementary Materials

The following are available online at https://www.mdpi.com/article/10.3390/toxics9090205/s1. **Figure S1.** Effect of PM exposure on cell mortality assessed using supravital propidium iodide (PI) staining and flow cytometry, as described in the Materials and Methods section, and expressed as a percentage of dead cells (brightly stained with PI) among all events. RAW 264.7 cells were exposed to NIST1648a or LAp120 at concentrations ranging from 0 to 100 μg/mL of culture medium (abscissa) for 6–72 h. #, ##, ###—*p* < 0.05, 0.01, 0.001, respectively, relative to cultures not exposed to NIST1648a (PM 0 μg/mL) at the same time point; $, $$, $$$—*p* < 0.05, *p* < 0.01, 0.001, respectively, relative to cultures not exposed to LAp120 (PM 0 μg/mL) at the same time point; and *, **, ***—*p* < 0.05, 0.01, 0.001, respectively, for cultures exposed to NIST1648a vs. LAp120 at the same concentration and time point. **Figure S2**. PM-induced late apoptosis assessed using supravital propidium iodide (PI) staining and flow cytometry and expressed as a percentage of apoptotic cells (dimly stained with PI) among all events. RAW 264.7 cell exposure conditions and significances as in Figure S1, **Figure S3.** PM-induced apoptosis assessed using propidium iodide (PI) staining of fixed cells and flow cytometry and expressed as a percentage of the sub-G1 events among all ones. RAW 264.7 cell exposure conditions and significances as in Figure S1. **Figure S4**. Effect of PM exposure on cell mortality assessed using the LDH test and expressed as a percentage of the values observed in control cultures not exposed to PM. RAW 264.7 cells were exposed to NIST1648a or LAp120 at concentrations ranging from 0 to 100 μg/mL of culture medium (abscissa) for 0–72 h. Significances as in Figure S1, **Figure S5.** Effect of PM exposure on ROS generation in viable cells at single cell level assessed using flow cytometry immediately after addition of PM at specified concentration to culture medium and after 1 and 4 h of exposure. Concentrations of NIST1648a or LAp120 (abscissa) were as in Figure S1. Values are expressed in relative fluorescence units (RFU). Significances as in Figure S1. **Figure S6**. Effect of PM exposure on cell number in cultures assessed using crystal violet staining and expressed as a percentage of the values observed in control cultures not exposed to PM. RAW 264.7 cell exposure conditions and significances as in Figure S4. **Figure S7**. Effect of PM exposure on oxidative burst assessed using the NBT reduction test and expressed as a percentage of the values observed in control cultures not exposed to PM. RAW 264.7 cell exposure conditions and significances as in Figure S4.
