# Supplementary material for: Metabolic Response of RAW 264.7 Macrophages to Exposure to Crude Particulate Matter and a Reduced Content of Organic Matter
Source: Toxics. 2021 Aug 30;9(9):205. doi: 10.3390/toxics9090205 (PMC8472964; doi:10.3390/toxics9090205)
Supplement: Supplementary file 1 [file toxics-09-00205-s001.zip › toxics-1318770-supplementary.pdf]

toxics

MDPI

Article

Supplementary Material: Metabolic Response of RAW264.7 Macrophages to Exposure to Crude Particulate Matter and a Reduced Content of Organic Matter

Monika Jankowska-Kieltyka, Adam Roman, Magdalena Mikrut, Marta Kowalska, Rudi van Eldik, Irena Nalepa

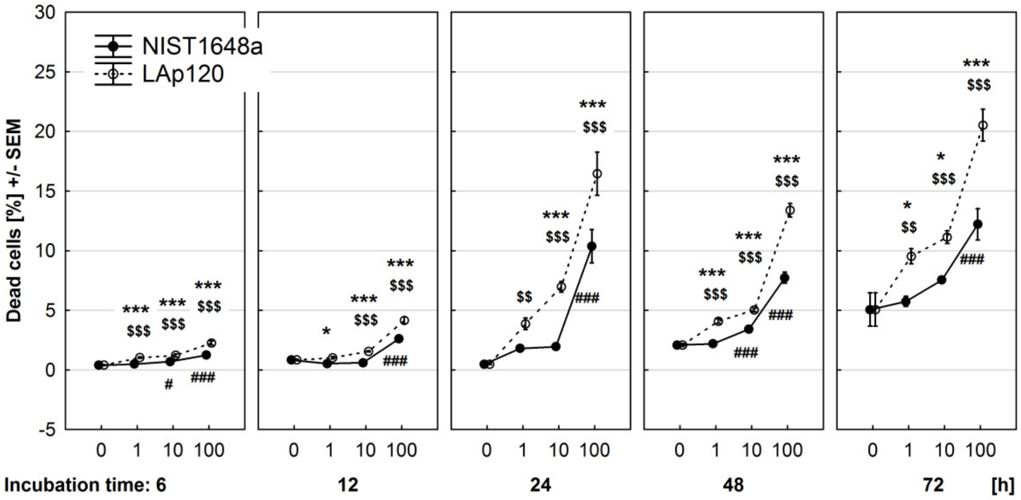

Figure S1. Effect of PM exposure on cell mortality assessed using supravital propidium iodide (PI) staining and flow cytometry, as described in Materials and Methods section, and expressed as % of dead cells (brightly stained with PI) among all events. RAW 264.7 cells were exposed to NIST1648a or LAp120 at concentrations ranging from 0 to 100 μg/ml of culture medium (abscissa) for 6–72 h. #, ##, ### $p < 0.05$, 0.01, 0.001 vs cultures not exposed to NIST1648a (PM 0 μg/ml) at the same time point. $, $$, $$$ $p < 0.05$, $p <$ 0.01, 0.001 vs cultures not exposed to LAp120 (PM 0 μg/mL) at the same time point. *, **, *** $p < 0.05$, 0.01, 0.001 cultures exposed to NIST1648a vs LAp120 at the same concentration and time point.

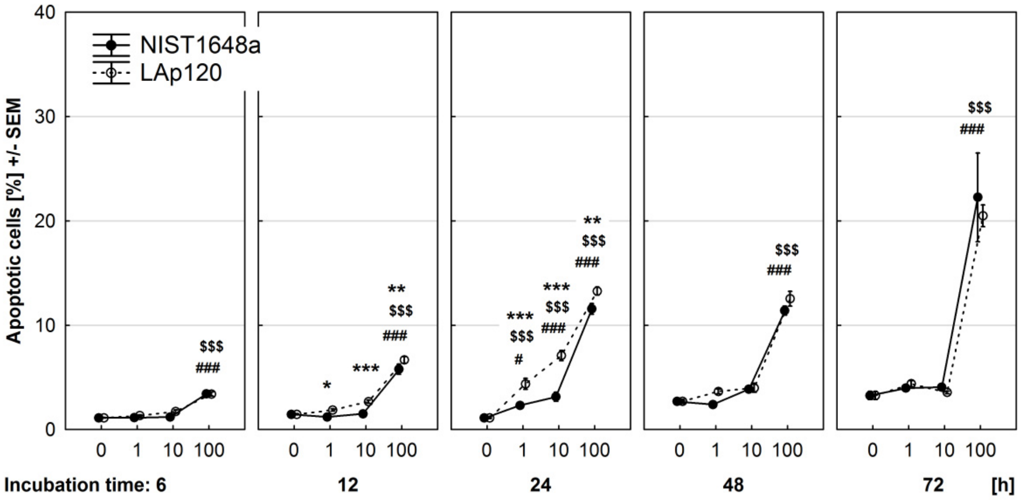

Figure S2. PM-induced late apoptosis assessed using supravital propidium iodide (PI) staining and flow cytometry and expressed as % of apoptotic cells (dimly stained with PI) among all events. RAW 264.7 cell exposure conditions and significances as in Figure S1.

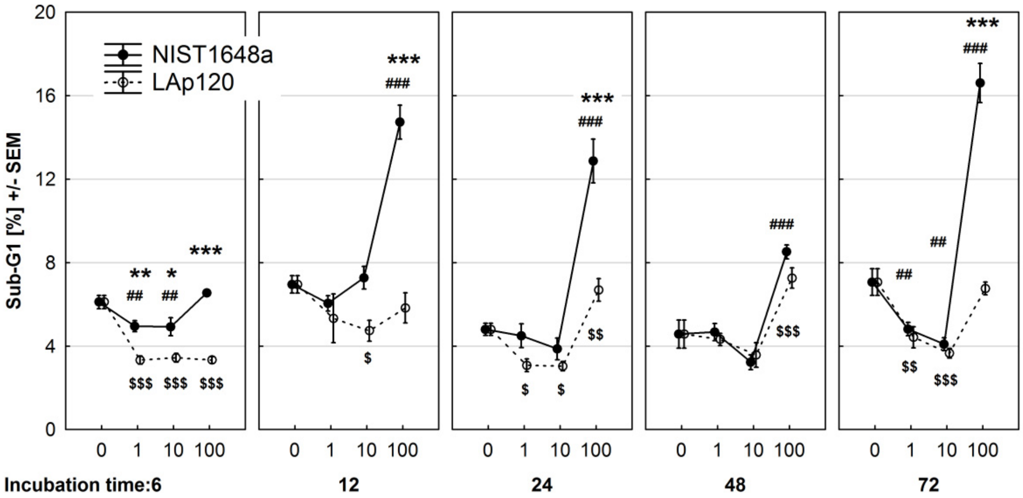

Figure S3. PM-induced apoptosis assessed using propidium iodide (PI) staining of fixed cells and flow cytometry and expressed as % of the sub-G1 events among all ones. RAW 264.7 cell exposure conditions and significances as in Figure S1.

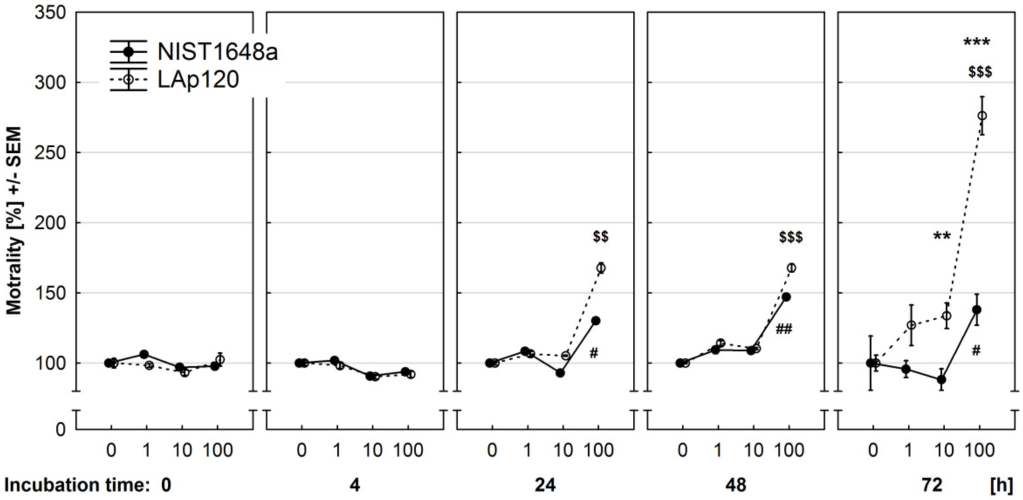

Figure S4. Effect of PM exposure on cell mortality assessed using the LDH test and expressed as % of the values observed in control cultures not exposed to PM. RAW 264.7 cells were exposed to NIST1648a or LAp120 at concentrations ranging from 0 to 100 μg/mL of culture medium (abscissa) for 0–72 h. Significances as in Figure S1.

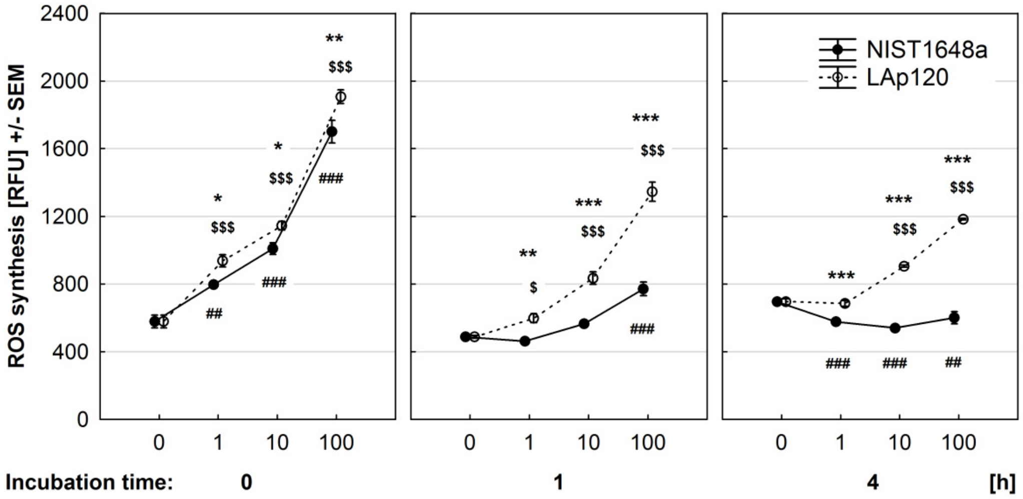

Figure S5. Effect of PM exposure on ROS generation in viable cells at single cell level assessed using flow cytometry immediately after addition of PM at specified concentration to culture medium and after 1 and 4 h of exposure. Concentrations of NIST1648a or LAp120 (abscissa) were as in Figure S1. Values are expressed as relative fluorescence units (RFU). Significances as in Figure S1.

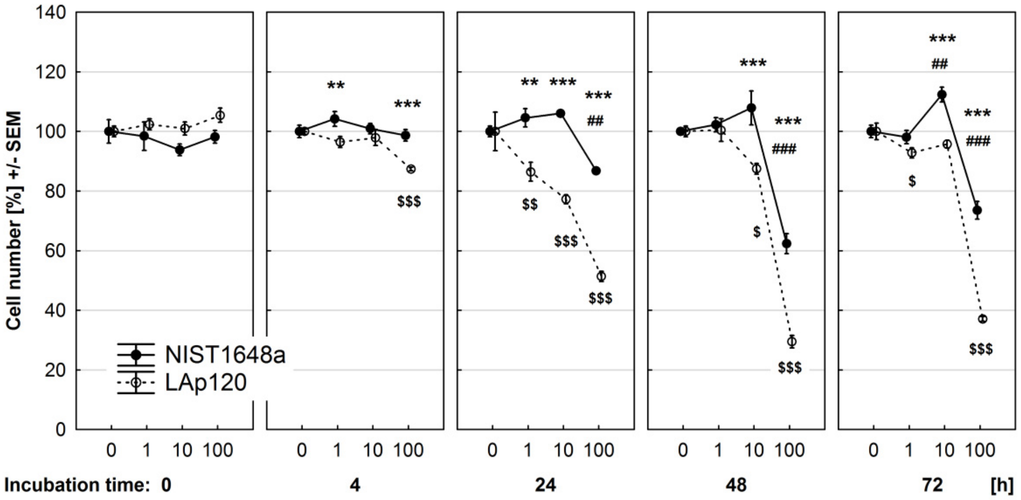

Figure S6. Effect of PM exposure on cell number in cultures assessed using crystal violet staining and expressed as % of the values observed in control cultures not exposed to PM. RAW 264.7 cell exposure conditions and significances as in Figure S4.

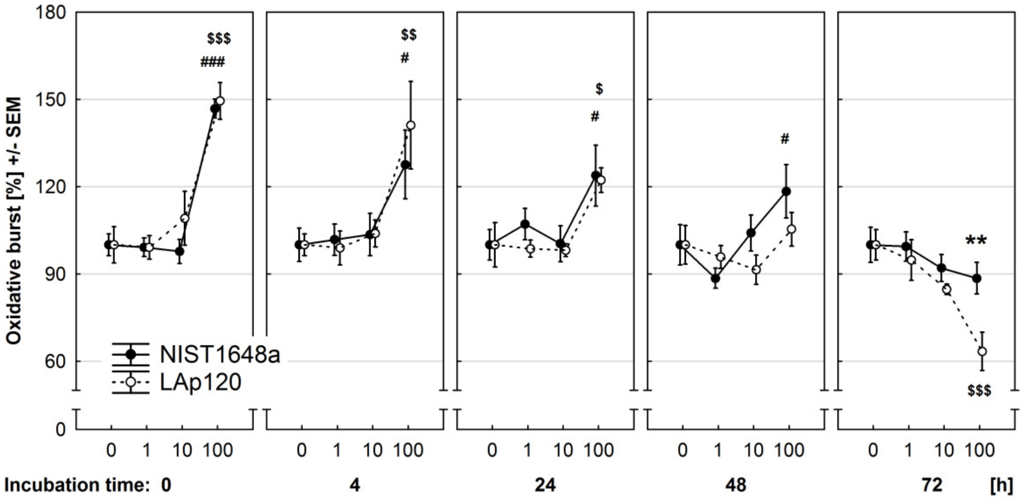

Figure S7. Effect of PM exposure on oxidative burst assessed using the NBT reduction test and expressed as % of the values observed in control cultures not exposed to PM. RAW 264.7 cell exposure conditions and significances as in Figure S4.
